# Orbital and Midface Reconstruction in a Case of Fibrous Dysplasia

**Published:** 2021-02-15

**Authors:** Megan L. Dietze-Fiedler, Katie Copeland, Matthew P. Fahrenkopf, Joshua P. Kelley, John J. Iacobucci

**Affiliations:** ^a^Michigan State University College of Human Medicine, Grand Rapids; ^b^Spectrum Health/Michigan State University Plastic Surgery Residency, Grand Rapids; ^c^Aesthetic Plastic Surgery of Grand Rapids, Grand Rapids, Mich

**Keywords:** fibrous dysplasia, midface, orbital, craniofacial, rib graft

## DESCRIPTION

The patient was a 33-year-old man with a medical history significant for autism and congenital agenesis of bilateral optic nerves who presented with progressive enlargement of the right malar region. Maxillofacial computed tomographic scan revealed bony hypertrophy involving the right inferolateral orbit, lateral maxilla, and zygoma consistent with fibrous dysplasia.

## QUESTIONS

1. What is the etiology of fibrous dysplasia?
2. What are the associated signs and symptoms of fibrous dysplasia?
3. What are the goals and methods for treating craniofacial fibrous dysplasia?
4. What methods were used in this case and what were the outcomes?

## DISCUSSION

Fibrous dysplasia is a relatively uncommon, benign condition in which normal bone is replaced by fibrous connective tissue and poorly formed trabecular bone. This occurs due to an activating mutation of the *GNAS1* gene that leads to excess production of interleukin 6.^1^ This then stimulates osteoclast differentiation and activity, resulting in excessive collagen production and deposition.^1^ The lesions typically begin in the bone marrow cavity and expand into surrounding cortical bone. Radiographically, this is seen as a lytic lesion with “ground glass” appearance, expansion of the involved area of bone, and potential distortion of bone shape.

Lesions most commonly occur in a single bone (monostotic) but may involve multiple bones (polyostotic). While fibrous dysplasia can affect any bone in the body, it most frequently involves the maxilla, mandible, and bones of the skull.^1^ The symptom profile depends on the location of the lesion. When the dysplasia leads to displacement of the orbit and globe, symptoms may include diplopia, proptosis, and eventual blindness from optic nerve compression. Onset and growth typically occur prior to 30 years of age, after which lesions become more stable.^2^ Malignant degeneration occurs in only approximately 0.5% of cases and is most common with polyostotic lesions. The typical malignancy is osteogenic sarcoma, but fibrosarcoma and chondrosarcoma have also been reported.^2^

The treatment goals are to eliminate or reduce symptoms and achieve aesthetic improvement. Radiotherapy is contraindicated for treatment due to the 400-fold increase in risk for malignant degeneration.^3^ Medical management with bisphosphonates has been shown to alleviate pain and improve radiological appearance; however, the mainstay of treatment is surgical. Surgical approaches may be either conservative or radical. The conservative approach involves curettage, contouring, or remodeling. This often requires reoperation due to recurrence. Initially, the conservative approach was thought to be beneficial in younger patients until tissue growth slowed. Alternatively, radical excision with reconstruction allows complete removal of the lesion and is less likely to have recurrence. The radical approach has become more widely accepted because of advances in surgical techniques and success of surgery without relapse.^1^

**Figure 1:**
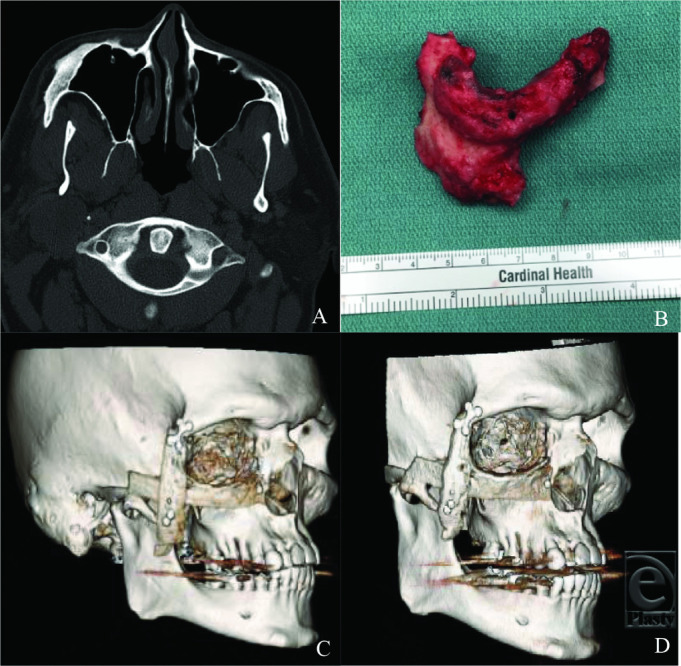
(*a*) Preoperative CT scan with right malar thickening from fibrous dysplasia. (*b*) Resected lesion including the right zygomatic arch, zygoma, inferior and lateral orbital wall, and lateral maxilla. (*c*) 3D maxillofacial CT on postoperative day 1. (*d*) 3D maxillofacial CT scan 1 year postoperatively. CT indicates computed tomography; 3D, three-dimensional.

In this patient, a surgical approach with staged reconstruction was done. The areas of involvement were identified as irregular, pinkish invasion of the bone and were resected. The resection included a portion of the zygomatic arch, the zygoma, inferior and lateral orbital wall, and the lateral maxilla on the right (Fig 1*b*). Temporary reconstruction was performed using titanium mesh and plates for the orbit and Medpor for the zygomatic arch and maxilla. After final pathology confirmed clear margins, a second surgery was performed for definitive reconstruction. The titanium plates, mesh, and Medpor were removed and replaced with autologous bone from fifth and sixth ribs. The autologous bone reconstruction with rib grafts provided an acceptable aesthetic result that remains stable (Fig 1*d*).

Craniofacial fibrous dysplasia often presents as facial deformity. In the fronto-orbito-malar region, treatment with excision and bone grafting can achieve aesthetically and functionally acceptable results. This case demonstrates that fibrous dysplasia involving the orbit and midface can be adequately treated with resection and staged reconstruction with autologous bone.
